# Revisiting the genetic support of leukotriene pathway drug targets in atherosclerotic cardiovascular disease

**DOI:** 10.3389/fgene.2026.1812905

**Published:** 2026-10-14

**Authors:** Tristan St-Laurent, Julien St-Pierre, Julien Quang Le Van, Marc-André Legault

**Affiliations:** 1 Faculté de pharmacie, Université de Montréal, Montreal, QC, Canada; 2 Centre de recherche Azrieli du CHU Sainte-Justine, Montreal, QC, Canada; 3 Montreal Heart Institute, Montreal, QC, Canada

**Keywords:** ALOX5, arachidonate 5-lipoxygenase, atherosclerotic cardiovascular disease, drug target validation, genetic epidemiology, leukotriene pathway

## Abstract

Arachidonate 5-lipoxygenase (5-LO) and its activating protein, encoded by *ALOX5* and *ALOX5AP* respectively, are two leukotriene pathway proteins suspected to play a role in atherosclerotic cardiovascular disease (ASCVD) through inflammatory mechanisms. Prior genetic association studies linking *ALOX5* and *ALOX5AP* to ASCVD have motivated clinical trials of 5-LO inhibitors, such as atreleuton, which was associated with reduced atherosclerotic plaque progression in patients with a recent acute coronary syndrome in a phase II trial. However, the effect of 5-LO inhibition on ASCVD clinical outcomes remains unclear. Using data from the UK Biobank, we revisit previously reported genetic associations of *ALOX5* and *ALOX5AP* with ASCVD using a multi-ancestry meta-analysis including data from 378,962 participants. We characterize ancestral differences in previously studied *ALOX5* and *ALOX5AP* polymorphisms which could have led to population stratification. When adequately accounting for ancestry, these previous associations were not replicated. In addition to our replication efforts, we used association scans and transcriptome-wide association studies to test the involvement of leukotriene pathway genes with ASCVD. Overall, our results do not support a role for genetic variation in *ALOX5* or its activating protein in ASCVD risk.

## Introduction

The management of low-density lipoprotein cholesterol levels is the cornerstone of secondary prevention of stable atherosclerotic cardiovascular disease (ASCVD) (Virani et al., 2020). However, the importance of managing the immune and inflammatory responses in the context of ASCVD is increasingly recognized. Recent results from randomized controlled trials showed that IL-1β blockers and colchicine, two agents that modulate inflammatory pathways, reduced major adverse cardiovascular events in individuals with a recent myocardial infarction (MI) (Ridker et al., 2017; Tardif et al., 2019). These results highlight the importance of inflammation as a promising therapeutic target that could reduce residual cardiovascular risk.

The leukotriene pathway is an inflammatory pathway that was previously linked to inflammation in the vascular wall and to the progression and instability of atherosclerotic plaque (Wang et al., 2023; Fredman et al., 2016). Genetic studies have identified variants in *ALOX5*, which encodes arachidonate 5-lipoxygenase (5-LO), that were associated with subclinical ASCVD (Dwyer et al., 2004), suggesting that 5-LO action may be relevant in ASCVD. Indeed, 5-LO inhibition targets an early step in leukotriene biosynthesis, and atreleuton, a 5-LO inhibitor, reduced atheroma progression in a phase II clinical trial, as well as epicardial adipose tissue in patients with recent acute coronary syndrome (Matsumoto et al., 2016; Almeida et al., 2020). Similarly, inhibiting the 5-LO activating protein, encoded by *ALOX5AP*, reduced MI-related biomarkers, including C-reactive protein, in a randomized trial (Hakonarson et al., 2005). However, the effect of medications targeting the leukotriene pathway on ASCVD remains uncertain as they have not been evaluated in cardiovascular clinical outcome trials. Moreover, genetic association studies have yielded inconsistent results regarding the association of 5-LO with ASCVD (Tsai et al., 2016). We briefly summarize the genetic evidence in Supplementary Table S1, which illustrates that previous genetic studies have reached conflicting conclusions regarding the involvement of 5-LO in ASCVD.

In this study, we revisited the association between ASCVD and variants in genes encoding the drug targets 5-LO and 5-LO activating protein, using a large-scale cohort of up to 378,962 participants from the UK Biobank and publicly available data from large genetics consortia (Bycroft et al., 2018). We used a trans-ancestral meta-analysis approach with the goal of replicating previous findings and identifying potentially novel genetic associations linking 5-LO to ASCVD. An interaction model in individuals with a high polygenic risk score of coronary artery disease, and a model of incident ASCVD in individuals with established ASCVD were also considered. Finally, a transcriptome-wide association study (TWAS) was conducted to assess the association between genetically predicted gene expression of 18 different leukotriene pathway genes and ASCVD.

## Methods

### UK Biobank

The UK Biobank is a population cohort of 500,000 individuals recruited in the United Kingdom (Bycroft et al., 2018). It includes genotype data on all participants and information on medical conditions obtained through questionnaires, verbal interviews with trained nurses and linkage with medico-administrative data repositories (e.g., hospitalization, death and cancer registries). The UK Biobank is publicly available for health researchers. All participants provided informed consent, and the UK Biobank has approval from the North West Multi-centre Research Ethics Committee (MREC) as a Research Tissue Bank (RTB). The current study was conducted under application #399545 and ethics approval for this project was obtained from the Research Ethics Committee of the CHU Sainte-Justine (2026–8,793). Supplementary Table S2 describes the data fields used to define covariates in our study.

#### ASCVD outcomes, biomarkers and proxy phenotypes of 5-LO activity

Following previous work, we algorithmically defined ASCVD outcomes based on linked diagnostic and surgical procedure codes from hospitalization records (Supplementary Table S3), including MI, ischemic stroke (IS), and revascularization procedures (percutaneous coronary intervention (PCI)/coronary artery bypass graft (CABG)) (Legault et al., 2022). We also included biomarkers of inflammation and atherosclerosis, including the carotid intima-media thickness (cIMT), LDL cholesterol (LDL-C), and C-reactive protein (CRP). Omega-6 levels were additionally considered as a candidate endophenotype of 5-LO activity in sensitivity analyses. Arachidonic acid, an omega-6 fatty acid, is a substrate of 5-LO and *ALOX5* variants have been associated with omega-6 fatty acid metabolism, motivating the use of this trait as a proxy of 5-LO activity (Stephensen et al., 2011). Circulating leukotriene levels were not measured in UK Biobank participants and thus could not be used as proxies of 5-LO activity.

#### Genotyping *ALOX5* and *ALOX5AP* variants

Previous studies have characterized the phenotypic effects of Sp1 repeat polymorphisms in the ALOX5 promoter (Dwyer et al., 2004; Stephensen et al., 2011; Hartiala et al., 2011; Gamme et al., 2016; Maznyczka et al., 2008; Todur and Ashavaid, 2012; González et al., 2007). Following this work, we first investigated the effects of this polymorphism on ASCVD in the UK Biobank. The Sp1 motif consists of tandem repeats of the 5′-GCGGGG-3′ sequence, and observed alleles in the ALOX5 promoter include between 1 and 11 copies of this motif. To genotype this tandem repeat, we relied on the whole genome sequencing based genotype calls (DRAGEN Whole Genome Sequencing [WGS] pVCF, UK Biobank field #24310) mapped to the human reference genome build GRCh38. The DRAGEN WGS release incorporates sample-level machine learning recalibration prior to joint variant calling, representing one of the leading approaches to variant calling and achieving SNV and INDEL precision exceeding 99.8% when assessed against Genome in a Bottle (The UK Biobank Whole-Genome Sequencing Consortium et al., 2025). No additional filtering beyond the pipeline’s default quality thresholds was applied to the Sp1 repeat calls. The Sp1 repeats were represented in the genotype calls as deletions or additions from which we calculated the number of repeats. In HGVS notation, the most common repeat number (5 copies) in our data is represented as NC_000010.11:g.45374100GCGGGG[5].

For *ALOX5AP*, we focused on a previously described four-SNP haplotype called “HapA” (Helgadottir et al., 2004). We used two different approaches to model the effect of this haplotype. First, for the recessive model, DRAGEN WGS pVCF (UK Biobank field #24310) was used to obtain the alleles defining HapA at chromosome 13 positions 30,725,416 (G), 30,737,959 (T), 30,752,304 (G), and 30,758,410 (A) using the GRCh38 reference. This dataset contains information on all UK Biobank participants, but does not provide phase information, which is not required to recover the recessive encoding of the haplotype; both chromosomal segments are necessarily identical in homozygotes. When testing additive effects, we used phased data to identify heterozygous carriers of HapA. Phased genotypes, estimated from WGS using SHAPEIT, were available for a subset of 200,000 participants (UK Biobank data-field #20279). The use of the larger sample size for the recessive encoding analysis maximizes power but may affect comparisons between the recessive and additive models, as differences could be due to power.

We conducted genetic quality control by excluding related individuals as reported in UK Biobank data field #22020. We also excluded individuals with discrepancies between self-reported (field #31) and genetically inferred sex (field #22001) as well as individuals with sex chromosome aneuploidies (field #22019). We used a trans-ancestral meta-analytic approach to account for population stratification, while maximizing sample size and the inclusion of participants, as described in the next section.

#### Trans-ancestral meta-analysis approach

UK Biobank participants self-reported their ethnicity using a touchscreen-based questionnaire (data field #21000). This questionnaire included the following options: White, British, Irish, Any other white background (grouped under European), Black or Black British, Caribbean, African (grouped under African), Indian, Pakistani, Bangladeshi (grouped under South Asian), and Chinese (under East Asian). We used self-reported ethnicity to interpret clusters obtained using Principal Component Analysis (PCA) of genotype data. Specifically, a random forest classifier was trained to predict self-reported ethnicity using the first 10 genetic principal components, similar to the method of global ancestry assignment performed by the Genome Aggregation Database (gnomAD) (Karczewski et al., 2020). Unrelated individuals with a prediction probability >99% were retained (Supplementary Figure S1, panel B). Finally, after observing significant overlap between ancestry groups on the PCA plot, we imposed a maximum distance (<95th percentile) from the ancestry cluster centroids on the two-dimensional projection to maximize homogeneity within our ancestry groups. The resulting ancestry groups are shown in Supplementary Figure S1, panel C, and the number of individuals under each genetically predicted ancestry group is reported in Table 1. Although the ancestry grouping methodology could result in some misclassification, we used an association method that accounts for population stratification via mixed effects modeling (REGENIE), reducing the risk of bias (Dias et al., 2025).

**TABLE 1:** Population characteristics stratified by genetically predicted ancestry group. Carotid intima-media thickness (cIMT) is measured in micrometers.

​	Genetically predicted ancestry group			
	African	East Asian	European	South Asian
N Total	7,704	1,738	362,392	7,128
Age (±SD)	51.9 ± 8.1	52.4 ± 7.6	56.8 ± 8.0	53.5 ± 8.5
Male (%)	3,296 (42.8%)	584 (33.6%)	167,261 (46.2%)	3,853 (54.1%)
N with cIMT measurement (%)	284 (3.7%)	151 (8.7%)	37,618 (10.3%)	377 (5.3%)
cIMT (±SD)	711.56 ± 133.49	659.14 ± 133.67	689.53 ± 127.61	667.66 ± 133.70
ASCVD outcomes				
Myocardial infarction - n (%)	225 (2.9%)	31 (1.8%)	19,244 (5.3%)	661 (9.3%)
Ischemic stroke - n (%)	183 (2.4%)	20 (1.2%)	8,563 (2.4%)	170 (2.4%)
Revascularization procedures - n (%)	111 (1.4%)	30 (1.7%)	17,203 (4.7%)	698 (9.8%)

### Statistical analyses

#### Replication of *ALOX5* and *ALOX5AP* genetic associations with ASCVD

Linear and logistic regression models adjusted for age, age^2^, sex, and the first 10 genetic principal components were used to test for genetic associations with continuous and binary outcomes, respectively, within ancestry groups, unless otherwise specified. We used R v4.4.0 (https://www.r-project.org/) to conduct the statistical analyses. We used a random-effects meta-analysis using the restricted maximum-likelihood estimator (R package metafor v4.8-0) to combine effect estimates from different ancestry groups (Viechtbauer, 2010) given observed heterogeneity in for cIMT (Cochran’s Q test; p = 0.005) and CRP (p = 0.021).

We used two different genetic association models to test the association of *ALOX5* variants with the previously defined outcomes. First, we sought to directly replicate findings from Dwyer et al. (2004) by using a binary coding where individuals carrying the major European allele of 5 repeats were treated as non-exposed, and any other genotype was treated as exposed. We refer to this model as the *homozygous non-5 repeats model* because only individuals with two copies of an alternative allele (with respect to the European allelic frequencies) are coded as exposed. Because our intention was to replicate previous findings from Dwyer et al., we only considered the alleles having 3, 4, 5, or 6 copies of the motif. As an example, an individual with 4 copies of the motif on one chromosome and 3 copies of the motif on the other would be coded as *homozygous non-5 repeats*, since both motif counts are not equal to 5. To test for dosage effects, we used a second approach using the total number of Sp1 repeats as a continuous variable (*dosage model*). For this coding, we set the reference (coded value of 0) to the ancestry-specific mode in the number of Sp1 repeats (8 in African individuals, and 10 for other ancestry groups). We used this coding so that negative values represent fewer repeats than the ancestry-specific reference, while positive values indicate more repeats than the reference. For example, an individual of European ancestry with 5 copies of the motif on one chromosome and 4 copies on the other would have a dosage of −1 according to this *dosage model*.

For *ALOX5AP* we considered additive, dominant, and recessive genetic models of the effect of the HapA haplotype. Previous studies by Helgadottir *et al.* found associations between ASCVD outcomes and HapA carriers in a dominant model (Helgadottir et al., 2004; Helgadottir et al., 2005), and we used the other model formulations as sensitivity analyses. In the recessive model, individuals with two copies of HapA were considered exposed, while in the dominant model, individuals with at least one copy of HapA were considered exposed. Finally, in the additive model every copy of HapA was assumed to have a constant effect (i.e., HapA copies have an additive effect).

#### Evaluating the effect of *ALOX5* and *ALOX5AP* in individuals at genetic risk of coronary artery disease

We tested whether ALOX5 and ALOX5AP variants (Sp1 motif repeats and HapA) have different effects depending on a person’s genetic risk of coronary artery disease (CAD) by including interaction terms with a CAD polygenic risk score (UK Biobank field #26227) in our models. Concretely, for *ALOX5*, we modeled MI, IS, and PCI/CABG using Sp1 repeat genotype (homozygous addition vs. homozygous deletion vs. ancestry-mode carrier coding) with a genotype-CAD PRS interaction term, using age, age^2^, sex, and the first 10 genetic principal components as covariates. For *ALOX5AP*, dominant, additive, and recessive models were tested with a genotype-CAD PRS interaction term with the same covariates.

#### Evaluating the effect of *ALOX5* and *ALOX5AP* in individuals with established ASCVD

Established ASCVD was defined using ICD9/ICD10 diagnostic codes, operation codes, or self-reported diagnoses at baseline (UK Biobank field #20002) recorded prior to the consent date (UK Biobank field #200). MI was identified from codes for acute and old MI; IS from codes for cerebral artery occlusion, thrombosis, embolism, and infarction; and PCI/CABG from procedure codes for coronary artery graft replacement and angioplasty. Full code lists are provided in Supplementary Table S3. An incident ASCVD event was defined as occurring after the UK Biobank consent date and is associated with a hospital or death record of an ICD9, ICD10, or operation code (Supplementary Table S3) dated at least 30 days after the event that defined the established ASCVD. For MI events, this window is consistent with the ICD10 distinction between acute (I21) and subsequent (I22) myocardial infarction, which applies a comparable 4-week threshold (World Health Organization, 2019). For established ASCVD defined by a prior revascularization procedure, the Fifth Universal Definition of Myocardial Infarction uses the same 30-day threshold to distinguish procedure-related MI from a new primary MI (Mills et al., 2026).

#### Genetic association testing of common variants in the ALOX5 and ALOX5AP regions

We used REGENIE v4.1 (Mbatchou et al., 2021) to test for the association between common variants in the *ALOX5* or *ALOX5AP* regions and MI, IS, or PCI/CABG and adopted a trans-ancestral meta-analysis approach. The regions were defined according to GENCODE version 47 (Mudge et al., 2024) with ±100 kb padding. The tested regions for *ALOX5* and *ALOX5AP* were respectively chr10:45,274,176-45,546,119 and chr13:30,613,478-30,864,425. Quality control was performed on the UK Biobank genotype calls in an ancestry-specific manner, using a sample and variant missing rate filter of 0.1, minor allele frequency >0.01, and Hardy-Weinberg disequilibrium threshold set dynamically based on sample size (Greer et al., 2024) using PLINK v2.0.0-a.6. LM (Chang et al., 2015). Step 1 of REGENIE was implemented using the genotype call variants passing quality control for each ancestry separately. Next, step 2 of REGENIE was performed with the TOPMed imputed genotypes to obtain genetic associations of variants in the *ALOX5* and *ALOX5AP* regions with each outcome. A fixed-effect inverse-variance weighted meta-analysis across ancestries was performed following the approach of large-scale trans-ancestral GWAS (e.g., pan-UKBB study) (Karczewski et al., 2024) and supported by the widespread sharing of genetic effects across ancestry groups (Verma et al., 2024). The use of a fixed-effect model also maximizes power to detect genetic associations. Only variants present in at least two ancestries were included in the meta-analyses.

#### Transcriptome-wide association study

TWAS can identify genes whose genetically predicted expression is associated with a phenotype. Because it aggregates variants into gene-level signals, TWAS can be more interpretable and better powered than single-variant analyses, offering a complementary approach to testing *ALOX5* and *ALOX5AP* associations with ASCVD. TWAS also uses summary statistics of genetic associations from large-scale consortia, providing an additional source of external evidence and maximizing the sample size. We therefore applied S-PrediXcan (Barbeira et al., 2018) to ASCVD outcomes, using PredictDB (https://predictdb.org/) models derived from GTEx to define expression prediction weights (GTEx Consortium, 2013) in whole blood, aorta and coronary artery. Genomic positions were lifted from GRCh37 to GRCh38 using the “liftover” Python package (https://github.com/jeremymcrae/liftover). These tissues were selected based on relevance to pathophysiology of ASCVD. Because CAD is a polygenic disease, we used the polygenicity-adjusted model to reduce the risk of false positives (Liang et al., 2024). We used summary statistics for CAD from the CARDIoGRAMplusC4D and UK Biobank meta-analysis in this analysis (Nelson et al., 2017). To obtain a list of relevant leukotriene pathway genes, we limited the TWAS results to genes found in MSigDB’s leukotriene metabolic process gene set *GOBP_LEUKOTRIENE_METABOLIC_PROCESS* contributed by The Gene Ontology Consortium (Aleksander et al., 2023).

To further evaluate whether the leukotriene pathway is associated with CAD, we performed a gene set enrichment analysis using the *fgsea* R package (Korotkevich et al., 2021). Only the most significant TWAS result was retained when multiple Ensembl identifiers could be mapped to the same gene identifier. Leukotriene pathway genes from our previous list were grouped to define the gene set, and all genes were ranked according to their TWAS z-score.

#### Rare variant associations

We considered large-scale studies of rare variant associations including the Genebass resource (https://app.genebass.org/) reporting gene-based associations from an exome-wide association analysis in 394,841 individuals from the UK Biobank. This data portal provides results from burden tests (using different variants sets), Sequence Kernel Association Test (SKAT) and optimal SKAT (Karczewski et al., 2022). As a second data source investigating rare variant association analysis, we used the All by All platform (https://allbyall.researchallofus.org) encompassing 3,602 phenotypes tested in 392,030 participants of the All of Us Research Program (Lu et al., 2026). This platform also provides burden, SKAT and SKAT-O results.

#### Statistical corrections for multiple testing

We used Bonferroni corrected significance thresholds to account for the multiple statistical testing. We established the significance thresholds separately for association models of *ALOX5* Sp1 motif repeats and *ALOX5AP* HapA haplotype. For *ALOX5*, two models were assessed (*homozygous non-5 repeats* and *dosage*) each with three ASCVD outcomes and four biomarkers. The two models were also tested in a model of ASCVD incidence in individuals with pre-established ASCVD. The number of tests, totaling 16, implies a significance threshold of p = 0.05/16 = 3.1 × 10^−3^. For *ALOX5AP*, three models were assessed (dominant, additive, and recessive) for the three ASCVD outcomes. Only the highest-powered and most significant model (i.e., recessive) was used to test for associations with the four biomarkers and for the test in individuals with established ASCVD. Thus, the total number of tests for *ALOX5AP* was 14 corresponding to a corrected threshold of p = 0.05/14 = 3.6 × 10^−3^.

For the region-based genetic association scans using REGENIE, we used a threshold of p = 1.2 × 10^−3^. This threshold is based on the simpleM procedure which uses principal component analysis to estimate the effective number of independent tests within a genetic region (Gao et al., 2008). For each gene region, the estimate of the effective number of independent statistical tests, M_eff_, was then used to determine a Bonferroni-adjusted threshold: p < 0.05/M_eff_. Pre-computed estimates of the number of tests accounting for 95% of the genotypic variance in gene regions were extracted from the *ExPheWAS* platform which uses the same UK Biobank genotype data (Legault et al., 2022) (M_eff_ = 29 for *ALOX5*, and M_eff_ = 13 for *ALOX5AP*, implying a threshold of p = 0.05/42 = 1.2 × 10^−3^). By using a M_eff_-based strategy for multiple testing correction, we apply a more liberal threshold for significance and maximize power.

When performing the TWAS, 18 unique genes were considered in three different tissues. Only genes with genetically regulated gene expression can be assessed in TWAS resulting in the inclusion of 30 different tissue-gene pairs. The resulting Bonferroni-corrected significance threshold was p = 0.05/30 = 1.7 × 10^−3^.

#### Minimal detectable effect size

We calculated the minimal effect size detectable with our sample size using the standard formulas (Rosner, 2006). We report the minimum effect size detectable by our study with 80% power at α = 0.05 for the cIMT measurement (unadjusted two sample Z-test) and for MI as a binary outcome, using ALOX5 Sp1 non-5 repeat allele frequency and MI prevalence (unadjusted two proportion Z-test). For this analysis, we used the same homozygous non-5 repeat carrier grouping as Dwyer et al. in the European ancestry group.

## Results

### Identification of Sp1 motif variants in the ALOX5 promoter region in the UK Biobank

We characterized differences in the distribution of Sp1 motif repeats across ancestry groups in the UK Biobank (Figure 1). The mode of the total number of Sp1 repeats was different between individuals of African ancestry (8 repeats) vs. other ancestries (10 repeats), while heterozygosity of the variant was highest in individuals of African ancestry. The observed distributions are consistent with previously reported allele frequencies, with the 5-repeat allele predominating in individuals of European ancestry and “deletion” alleles being substantially more frequent in individuals of African ancestry (Dwyer et al., 2004; Hartiala et al., 2011; Assimes et al., 2008; Lima et al., 2006). Since the prevalence of ASCVD (Gebreab et al., 2015; Patel et al., 2021) and mean cIMT (Manolio et al., 1995; Whincup et al., 2012; Vriend et al., 2024) also vary between ancestry groups, these results suggest that residual population stratification could have led to false positives in previous studies investigating the effect of *ALOX5* Sp1 repeats (Table 1). As previous studies have classified individuals as carrying “deletions” or “additions” when compared to the mean of 10 repeats (Dwyer et al., 2004), we also report the proportion of individuals falling in these categories by ancestry (Figure 1, panel C) and show that this classification does not adequately capture genetic diversity at the locus in non-European populations.

**FIGURE 1:**
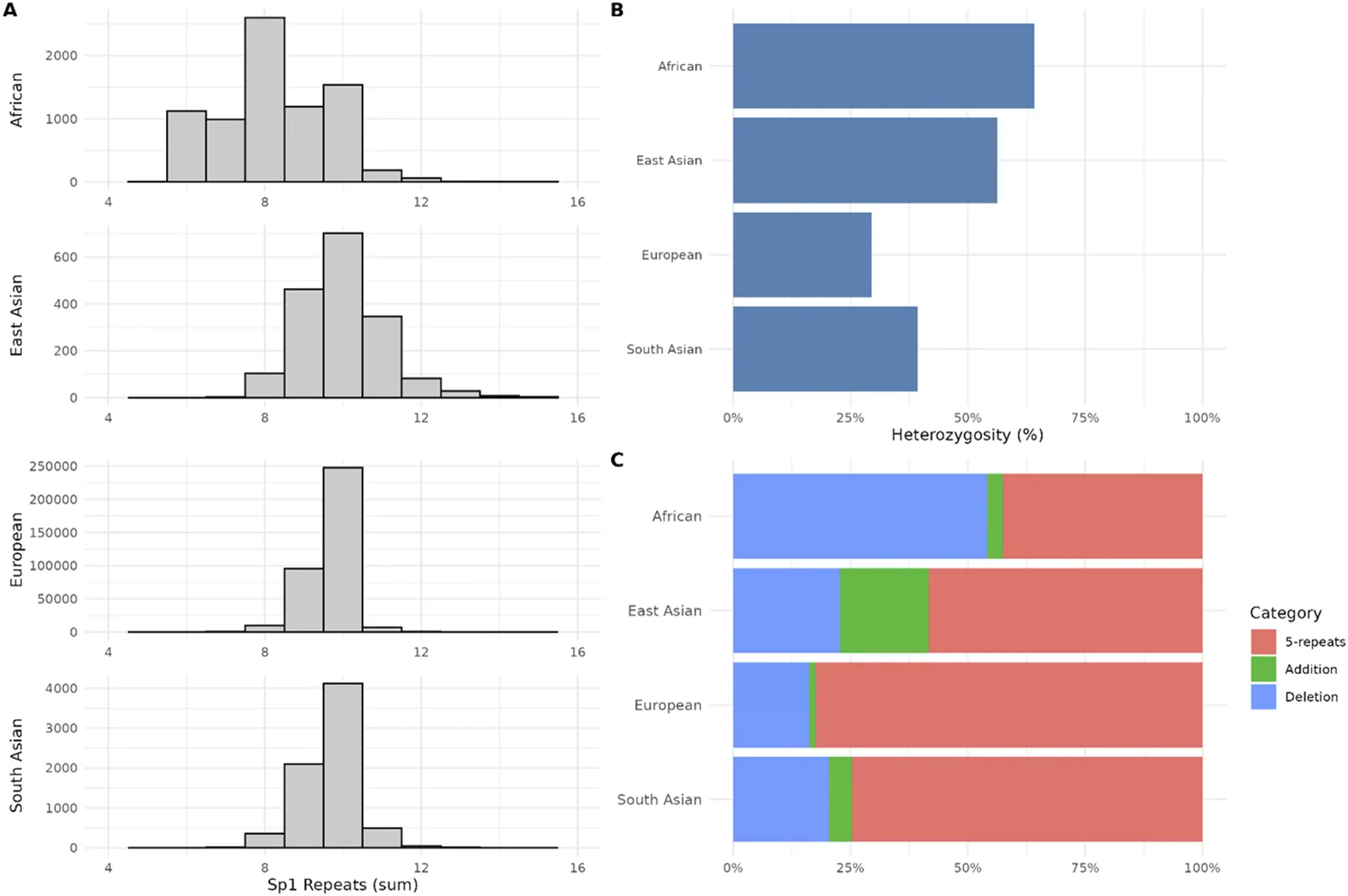
Overview of the ancestral diversity in the number of total ALOX5 promoter Sp1 repeat counts (both chromosomes). **(A)** Distribution of the number of repeats. **(B)** Sp1 repeat zygosity. **(C)** Proportion of Sp1 deletions and additions with respect to the most common genotype in individuals of European ancestry (10 repeats of the motif).

### Replicating the association of ALOX5 promoter Sp1 motif variants with cIMT and cardiovascular outcomes

Dwyer et al. (2004) showed that the number of Sp1 repeats in the promoter of *ALOX5* was associated with cIMT and CRP levels in a cohort of 470 participants from the Los Angeles Atherosclerosis Study. We sought to replicate the findings from this landmark study linking *ALOX5* to ASCVD using the same genetic association model (referred to here as the *homozygous non-5 repeats model*, see Methods). Although our results for cIMT were directionally concordant with this prior study in the European ancestry subgroup, our genetic effect estimates of *ALOX5* variants were not statistically significant for CRP, cIMT, LDL-C, MI, IS, or PCI/CABG (Supplementary Table S4). However, we observed a significant association between the Sp1 motif variant and CRP in the subgroup of individuals of African ancestry (p = 0.003). We treat this result as a subgroup finding that would require further validation, especially since there was no significant effect in the trans-ancestral analysis or the other subgroups. Finally, there was a non-significant increase in the candidate endophenotype of plasma omega-6 levels in individuals with two variant alleles (p = 0.06).

Given the null results for cIMT, we estimated the minimum detectable effect size. The model had 80% power to detect a significant effect of approximately 8 µm in the European ancestry group. Dwyer *et al.* reported mean effect differences of over 62 µm in their adjusted models between homozygous carriers of non-5 repeats alleles and individuals without two non-5 repeats alleles (Dwyer et al., 2004). Thus, our study had power to detect a substantially smaller effect (approximately 99.9% power to detect a four-fold smaller effect). For MI, using the same homozygous non-5 repeat carrier grouping in the European ancestry group alone, the model had 80% power to detect an OR of 1.30.

To test whether each additional Sp1 repeat has a constant effect, we fit an ancestry-specific dosage model of the ALOX5 promoter repeat, coding copy number relative to the most common allele in each ancestry group (reference = 0; Figure 1), with deletions as negative integers and additions as positive. In this analysis, the negative association between the plasma omega-6 and Sp1 number was significant (p = 8.90 × 10^−5^, Figure 2) and was directionally concordant between ancestries. Despite this association with a candidate proxy of 5-LO activity, omega-6 fatty acids being the main substrate of 5-LO, our results did not support an association between the dosage of Sp1 motif repeats and CRP, LDL-C, cIMT or any ASCVD clinical outcome (Figure 2).

**FIGURE 2:**
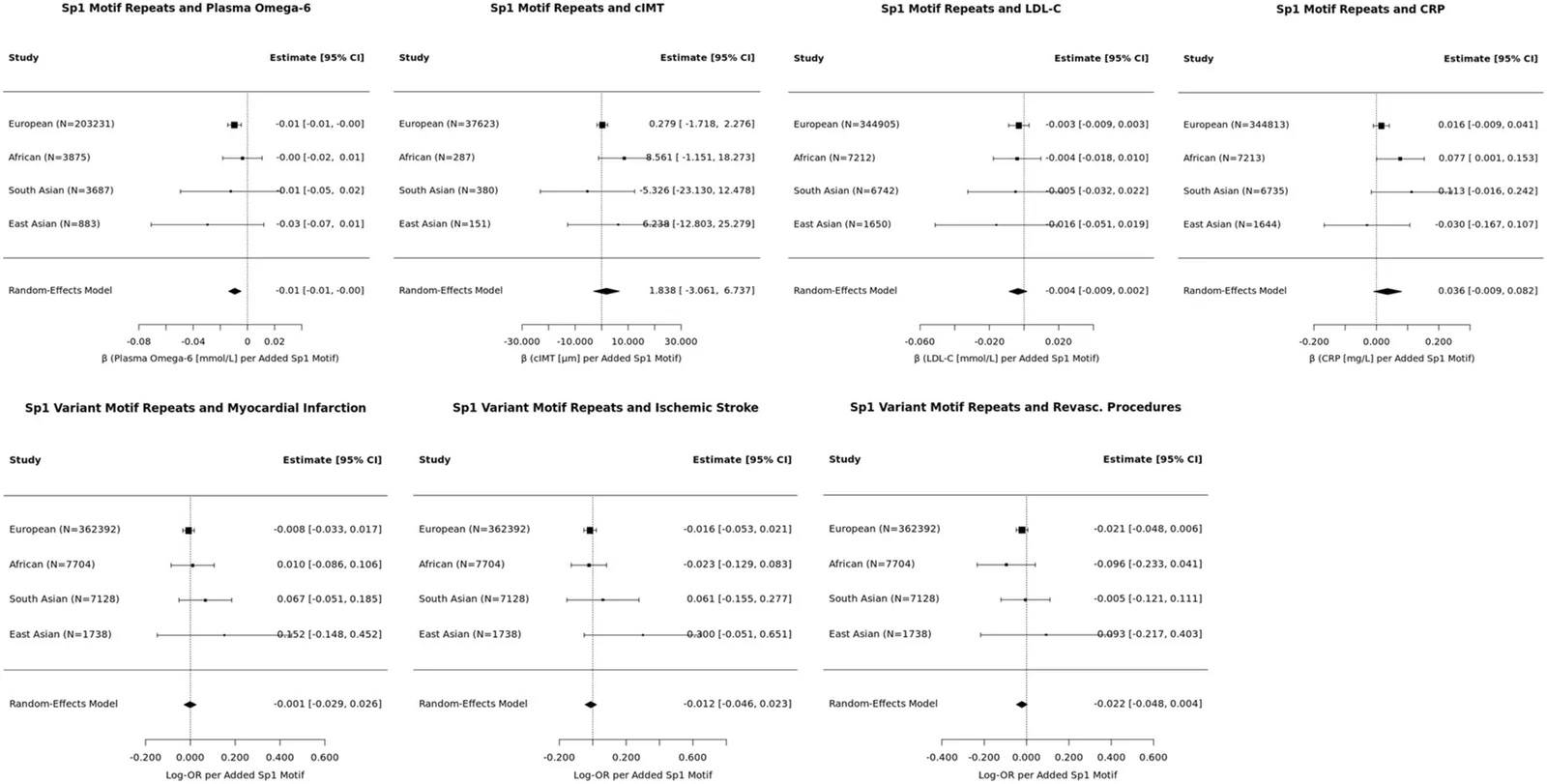
Effect of continuous dosage of *ALOX5* promoter Sp1 motifs on biomarkers and cardiovascular outcomes in the UK Biobank. Ancestry-specific effects are combined using a random-effects meta-analysis. Regression models are adjusted for age, age^2^, sex, and 10 genetic principal components.

### Replicating the association of ALOX5AP HapA with cardiovascular outcomes

*ALOX5AP*, encoding 5-LO activating protein, an essential cofactor for 5-LO activation, has previously been associated with ASCVD in genetic studies (Gamme et al., 2016; Helgadottir et al., 2004; Helgadottir et al., 2005; Lõhmussaar et al., 2005; Girelli et al., 2007; Crosslin et al., 2009; Burdon et al., 2010; Sharma et al., 2013; Yang et al., 2014). However, these findings have been inconsistently replicated. We therefore examined whether the *ALOX5AP* HapA haplotype is associated with ASCVD outcomes and with biomarkers of inflammation and atherosclerosis in our cohort. First, we characterized the frequency of the HapA haplotype in individuals of different ancestries and observed substantial ancestral variability (Figure 3). For instance, as previously described, we found that rs17222814, a SNP forming HapA, is monomorphic in the East Asian ancestry group (Zhang et al., 2006).

**FIGURE 3:**
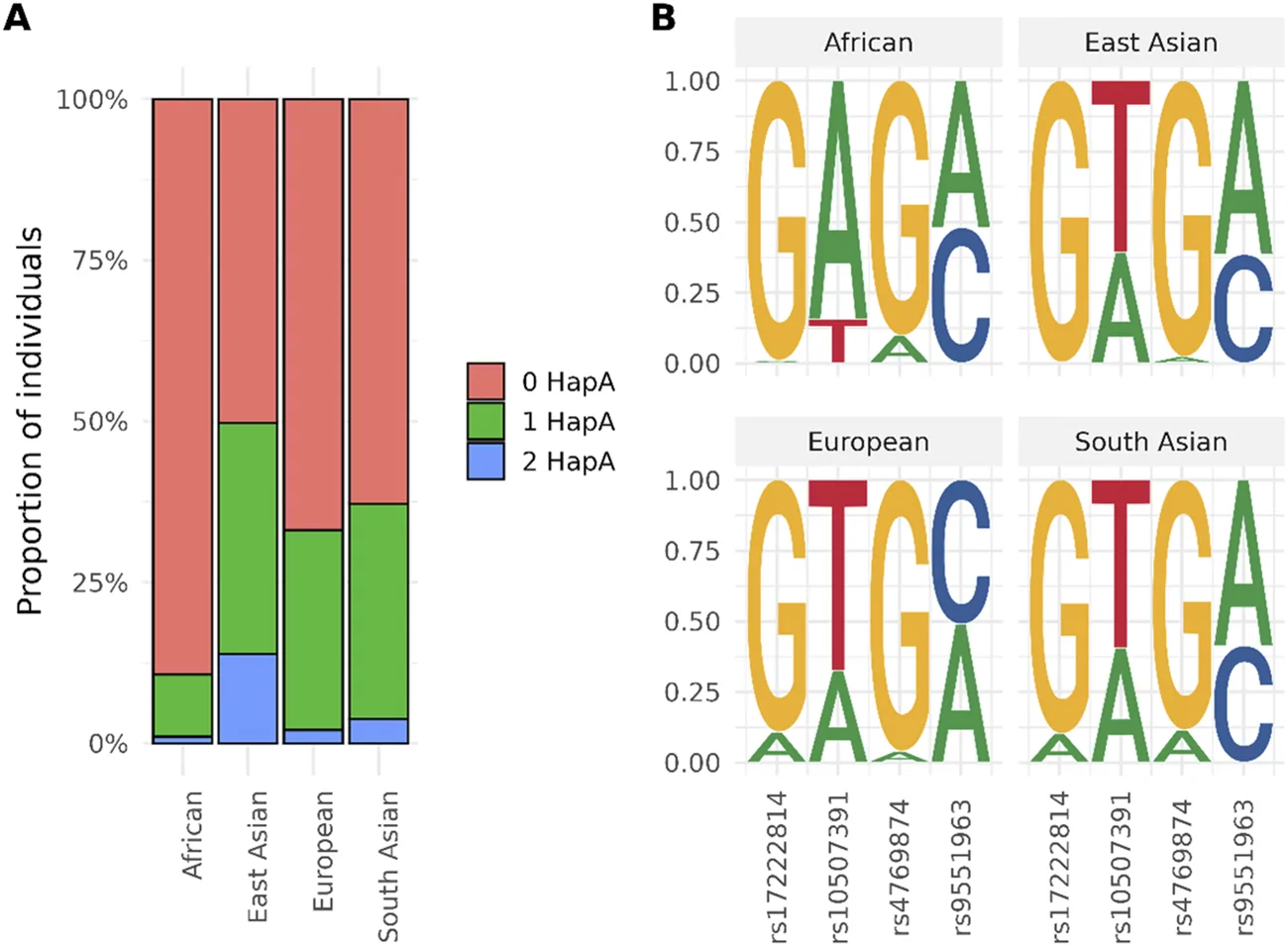
HapA genotype and allele frequencies of the variants forming HapA in different populations. **(A)** HapA genotype among different ancestries. **(B)** Allele frequency at the SNPs defining HapA across different ancestries, with rs17222814 at position 30,725,416, rs10507391 at position 30,737,959, rs4769874 at position 30,752,304, and rs9551963 at position 30,758,410 (GRCh38). HapA is defined as the “GTGA” genotype. The y-axis of panel B corresponds to information content.

Carriers of HapA (i.e., a dominant model) were not at significantly increased risk of any ASCVD outcome, thus the current analysis could not replicate previous findings by Helgadottir et al. (2004) (Supplementary Figure S2). As sensitivity analyses, other models were tested. First, dosage of HapA copies (i.e., an additive model) was not associated with any ASCVD outcome (Supplementary Figure S3). In contrast to the two prior models that had to use phased data only available for a subset of 155,933 participants, a recessive model evaluating homozygous carriers of HapA leveraging data from 378,962 participants was used and revealed a nominally significant increased risk of MI in the European-ancestry subgroup only (OR = 1.12 [1.02, 1.24], p = 0.02, Figure 4). This association did not remain significant after applying a Bonferroni correction accounting for the number of statistical models and outcomes assessed (see *Statistical corrections for multiple testing* in Methods). Additionally, HapA was not associated with the other considered ASCVD outcomes or biomarkers (Supplementary Table S5).

**FIGURE 4:**
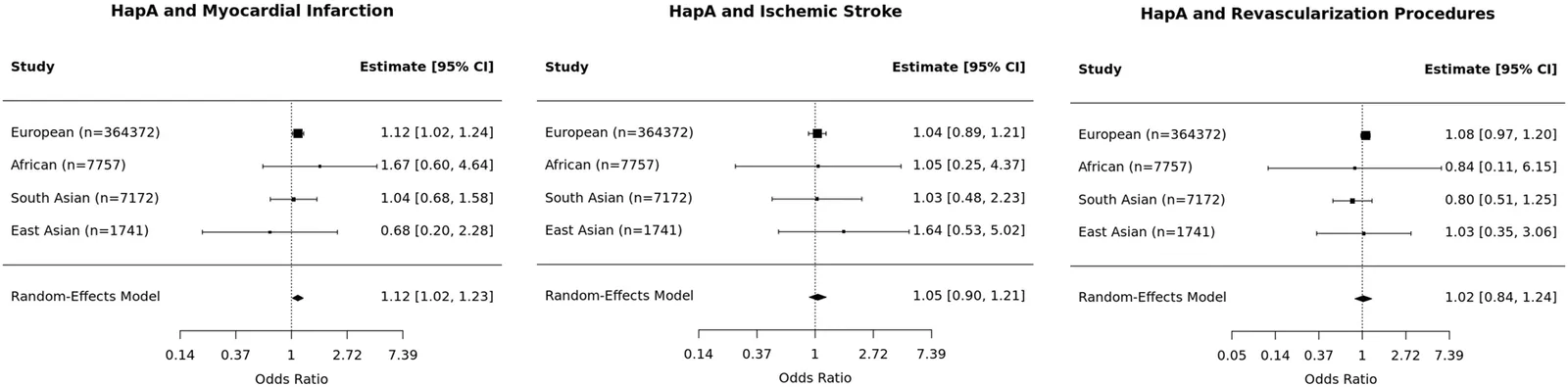
Effect of HapA on ASCVD outcomes in the UK Biobank using a recessive model. Ancestry-specific effects are combined using a random-effects meta-analysis. Regression models are adjusted for age, age^2^, sex, and 10 genetic principal components. HapA is a haplotype consisting of SNPs at four positions: rs17222814 [G], rs10507391 [T], rs4769874 [G], and rs9551963 [A].

### Effect of ALOX5 and ALOX5AP in individuals with established ASCVD

The leukotriene pathway may be involved in plaque stability after the initiation of ASCVD (Cipollone et al., 2005; Qiu et al., 2006). To test whether genetic variation in this pathway influences outcomes in high-risk individuals, we examined the effect of *ALOX5* and *ALOX5AP* variants in participants at high genetic risk of CAD. We did not find a statistically significant interaction between the *ALOX5* or *ALOX5AP* variants and a CAD PRS on their effect on ASCVD outcomes (results not shown). These results suggest that there is no effect heterogeneity of the considered variants on ASCVD across strata of genetically predicted CAD risk.

Genetically predicted CAD risk may not adequately capture individuals with established ASCVD. Using a complementary approach, we focused on individuals with evidence of prevalent ASCVD documented at the UK Biobank baseline visit and tested the association of *ALOX5* and *ALOX5AP* with incident ASCVD outcomes in this population. Of the 24,188 individuals of European ancestry with at least one prior documented or self-reported ASCVD event, 5,862 had an incident event after baseline, and the incidence of ASCVD in this group was not associated with the *ALOX5* promoter Sp1 motif under any of the considered models. ASCVD incidence was likewise not associated with HapA under our highest-powered recessive association model (Supplementary Table S6).

### Regional genetic association study to identify ALOX5 or ALOX5AP variants associated with ASCVD outcomes

Most previous studies of *ALOX5* and *ALOX5AP* focused on characterizing specific haplotypes or promoter variants (i.e., the *ALOX5AP* HapA and the Sp1 motif variant of *ALOX5*). Given the large number of high-quality imputed genetic variants in the UK Biobank, we sought to identify new genetic variants in the *ALOX5* or *ALOX5AP* gene regions that could be associated with ASCVD (Supplementary Figure S4; Supplementary Figure S5). Five *ALOX5* variants reached the statistical significance threshold in the discovery analysis for MI, but three of the five were not significant in the independent cohort from CARDIoGRAMplusC4D 1000 Genomes-based GWAS for MI (Nikpay et al., 2015). The two remaining variants were not present in the other dataset and therefore could not be replicated (Supplementary Table S7).

### Evaluation of *leukotriene* pathway genes using TWAS

In our TWAS based on data from 113,937 individuals with CAD from a large-scale GWAS meta-analysis, none of the 18 included leukotriene pathway genes with available data were significantly associated with CAD (Supplementary Table S8). Additionally, the gene set enrichment analysis of leukotriene pathway genes ranked by their TWAS z-score from whole blood tissue revealed no significant enrichment for CAD (normalized enrichment score = 0.76, p = 0.77).

### Rare variant association testing

Genetic support for drug targets can come from diverse data sources including common variant associations (e.g., from GWAS) or rare variant association analysis (Minikel et al., 2024). To complement our common variant-based analyses, we used the Genebass and All by All data portals to investigate the association of rare *ALOX5* and *ALOX5AP* variants with ASCVD. No significant associations were found with ASCVD-related phenotypes in this analysis (Supplementary Table S9).

## Discussion

Given the conflicting reports between leukotriene pathway genes and ASCVD, we revisited the association between ASCVD and key enzymes in this pathway, 5-LO and its activating protein (encoded by *ALOX5AP*), using a trans-ancestral meta-analysis approach in participants from the UK Biobank. To support our findings, we conducted TWAS analyses using summary statistics from large GWAS meta-analyses. Furthermore, we conducted association scans to identify new *ALOX5* or *ALOX5AP* genetic variants associated with ASCVD. In line with the hypothesis that the leukotriene pathway may be involved in the inflammatory response after atherosclerosis is established, we also considered associations in individuals across genetically predicted risk of CAD or that had evidence of prevalent CAD. Overall, despite our high statistical power to replicate associations reported in prior studies, our results do not support an association of genetic variants in *ALOX5* or *ALOX5AP* with ASCVD outcomes, cIMT, or C-reactive protein levels.

Repeats of Sp1-binding motifs in the *ALOX5* promoter region (Hoshiko et al., 1990) affect gene transcription (In et al., 1997) and were reported to be associated with carotid intima-media thickness in a cohort of 470 participants (Dwyer et al., 2004). Replication studies have since yielded conflicting results on the association of these variants with cardiovascular disease endpoints (Hartiala et al., 2011; Gamme et al., 2016; Todur and Ashavaid, 2012; González et al., 2007; Assimes et al., 2008; Maznyczka et al., 2007). Considering these conflicting findings, we sought to revisit the involvement of 5-LO in ASCVD using data from the UK Biobank, a large-scale genetic cohort of 500,000 participants (Bycroft et al., 2018). We found that the distribution of Sp1 motif counts as well as heterozygosity varied substantially across subgroups defined based on genetic ancestry, calling into question results from previous studies that used the 5-motif count as a reference point in ancestrally diverse cohorts (Dwyer et al., 2004; Sun et al., 2000). We hypothesize that residual population stratification could have led to false positive findings in previous studies since ASCVD risk and the distribution of biomarkers such as cIMT vary significantly across ancestries (Manolio et al., 1995; Whincup et al., 2012; Vriend et al., 2024). In the present study, we modeled the Sp1 repeat polymorphism using an ancestry-specific dosage which better accounts for these differences in allele frequencies across populations. Using circulating omega-6 as a candidate proxy of 5-LO activity, we observed that the number of *ALOX5* promoter Sp1 motifs was negatively associated with plasma omega-6. Arachidonic acid, one of the most abundant phospholipid fatty acids in plasma (Wu et al., 2014), is a substrate of 5-LO. We anticipate that a reduction in 5-LO activity would lead to the accumulation of arachidonic acid, providing a rationale for omega-6 fatty acids as a proxy phenotype of 5-LO activity. A few studies reporting associations between *ALOX5* variants and omega-6 fatty acids have been published to date (Dwyer et al., 2004; Stephensen et al., 2011; Allayee et al., 2008). Despite this analysis, we did not find significant associations between the number of *ALOX5* promoter repeats and ASCVD outcomes or related biomarkers. Although the findings were null, the complexity surrounding *ALOX5* transcriptional regulation and the Sp1 tandem repeats in the promoter should be highlighted. First, the effect of one or two motif deletions has been shown to both increase (Allayee et al., 2008) *ALOX5* transcription, and decrease (In et al., 1997; S et al., 1998) *ALOX5* promoter activity depending on the study, with different cell types being used to assess the effect of Sp1 motif variants on *ALOX5* gene expression (González et al., 2007; In et al., 1997; Allayee et al., 2008; S et al., 1998). Presumably, the effect of variants could vary based on cellular context, with DNA methylation likely playing a key role as demonstrated by earlier functional studies showing that promoter methylation distinguishes cells that produce 5-LO from those that do not (Uhl et al., 2002).

There are also conflicting reports regarding the association of the 5-LO activating protein (*ALOX5AP*) and ASCVD. For instance, Helgadottir *et al.* found that the four-SNP haplotype HapA was associated with MI in an Icelandic cohort of 1,403 unrelated individuals (779 cases), and with stroke in two independent cohorts (Helgadottir et al., 2004; Helgadottir et al., 2005). However, a subsequent meta-analysis of 30 studies found no association between HapA and ischemic stroke (Zheng et al., 2019), while another study also estimated a null effect on coronary heart disease (Huan et al., 2010). In our trans-ancestral meta-analysis, HapA was not associated with MI, IS, or revascularization procedures. There was, however, a modest association between HapA and MI (p = 0.02) in individuals of European ancestry, but no association with related ASCVD outcomes or biomarkers including CRP, LDL-C, and cIMT. As opposed to our *ALOX5* analysis, we did not find any association between *ALOX5AP* HapA and omega-6, which was used as a candidate proxy phenotype of 5-LO activity because arachidonic acid is a substrate of the enzyme. Given the lack of supporting evidence from other ancestries or outcomes, it is possible that the association between HapA and MI is spurious, and we interpret this result with care.

The strength of our approach lies in the large sample size totaling 378,962 individuals from four ancestry groups, as well as in the detailed characterization and careful modeling of ancestral differences in *ALOX5* and *ALOX5AP* allelic frequencies. We considered well-known variants that have been previously studied in replication efforts, and we used association scans and TWAS as hypothesis-free tests of association. Although there were a few significant hits in the regional analysis of *ALOX5* and MI, they could not be externally replicated, and considering the candidate gene approach, are likely false positives. We also tested the hypothesis that 5-LO or ALOX5AP inhibition could be associated with ASCVD outcomes after the initiation of atherosclerosis but found no significant association. Finally, we considered the possibility of rare variants in *ALOX5* and *ALOX5AP* being associated with relevant outcomes with burden and SKAT tests showing no association in both the UK Biobank and All of Us cohorts.

Notable methodological limitations of our analysis are the absence of some other ancestry groups which were not available through self-reporting (e.g., Middle Eastern), and the lack of leukotriene pathway specific molecular markers to serve as proxies for 5-LO and ALOX5AP activity (e.g., leukotriene metabolites). Our analysis does not rule out that multiple genes in the leukotriene pathway may influence ASCVD risk through gene-gene interactions, nor the possibility of nutrient-gene interactions driving ASCVD risk through arachidonic acid availability, or contexts of decreased 5-LO/ALOX5AP action driving arachidonic acid to clinically relevant alternate fates (e.g., thromboxane and prostaglandins *via* cyclooxygenases). Indeed, some studies have described significant leukotriene pathway variant interactions with omega-6 and omega-3 metabolism (Stephensen et al., 2011; Allayee et al., 2008; Hartiala et al., 2012), and a clinical study of patients with asthma observed increased spontaneous platelet aggregation following treatment with Zileuton, a 5-LO inhibitor, supporting the hypothesis that inhibiting 5-LO could redirect some arachidonic acid to the cyclooxygenase pathway (Wu et al., 2003). The cysteinyl leukotriene receptors or enzymes downstream of 5-LO may be more viable potential targets in this context due to plausibly reduced interaction with the cyclooxygenase pathway and sparing of 5-LO-mediated lipoxin production, which may be beneficial in the context of ASCVD (Chen et al., 2023). Furthermore, the association between ASCVD and 5-LO may be dependent on the localization of its activity within the plaque environment (Cipollone et al., 2005; Qiu et al., 2006). Currently, TWAS approaches may lack the resolution to detect highly localized effects, especially when relying on existing atlases of bulk gene expression in cohorts that do not necessarily have ASCVD.

In conclusion, neither hypothesis-driven replication analyses nor agnostic association scans support an association between common or rare *ALOX5* and *ALOX5AP* variation and ASCVD risk. Studies incorporating precise molecular measurements of leukotriene pathway products may provide more direct evidence on the impact of genetic variation in these genes.

## Data Availability

The data analyzed in this study is subject to the following licenses/restrictions: Access to UK Biobank data is available to approved researchers conducting health-related research in the public interest. Requests to access these datasets should be directed to https://www.ukbiobank.ac.uk/use-our-data/.
