# Supplementary material for: Revisiting the genetic support of leukotriene pathway drug targets in atherosclerotic cardiovascular disease
Source: Front Genet. 2026 Oct 14;17:1812905. doi: 10.3389/fgene.2026.1812905 (PMC13713012; doi:10.3389/fgene.2026.1812905)
Supplement: Supplementary file 1 [file DataSheet1.pdf]

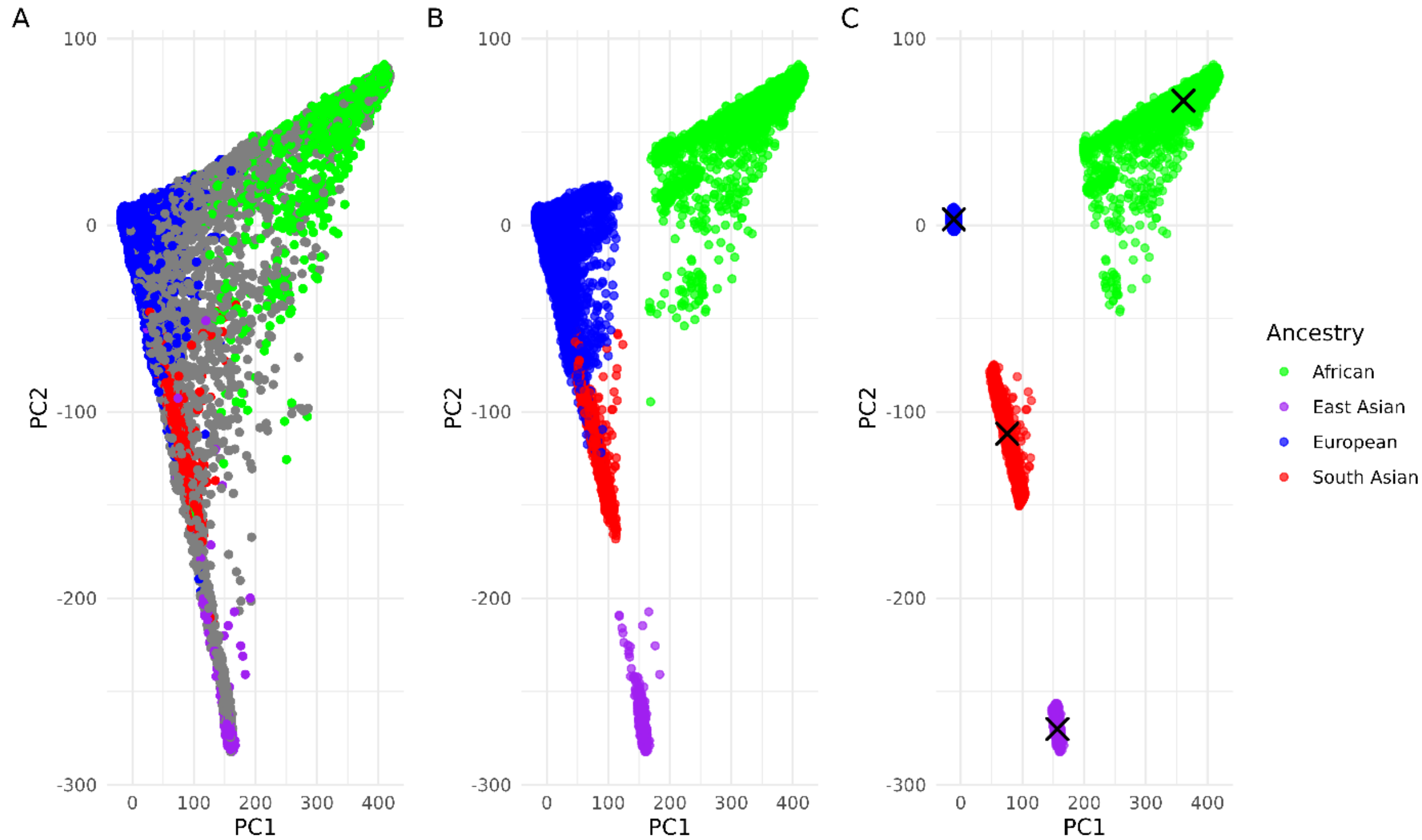


***Supplementary Figure 1. Principal component plots with self-reported and inferred ancestry***. ***A)*** *Projection of the individuals according to the first two PCs with colors representing self-reported ancestry;* ***B)*** *Predicted genetic ancestry based on a random forest classifier. All individuals are represented despite the confidence of the prediction;* ***C)*** *Projection of final, homogeneous ancestry groups. The first 2 principal components are plotted for each individual whose genetic ancestry could be predicted with at least 99% confidence by a random forest classifier trained on the first 10 genetic components to predict UK Biobank self-reported ethnicity. Centroids were defined (depicted by 'X's) for each continental genetic ancestry group and individual's whose distance from their corresponding centroid was greater than the 95$^{th}$ percentile were filtered out. In other words, the 5% of individuals who were most genetically distinguishable for each ancestry group based on the first 2 genetic principal components were filtered out to retain genetically homogenous groups.*

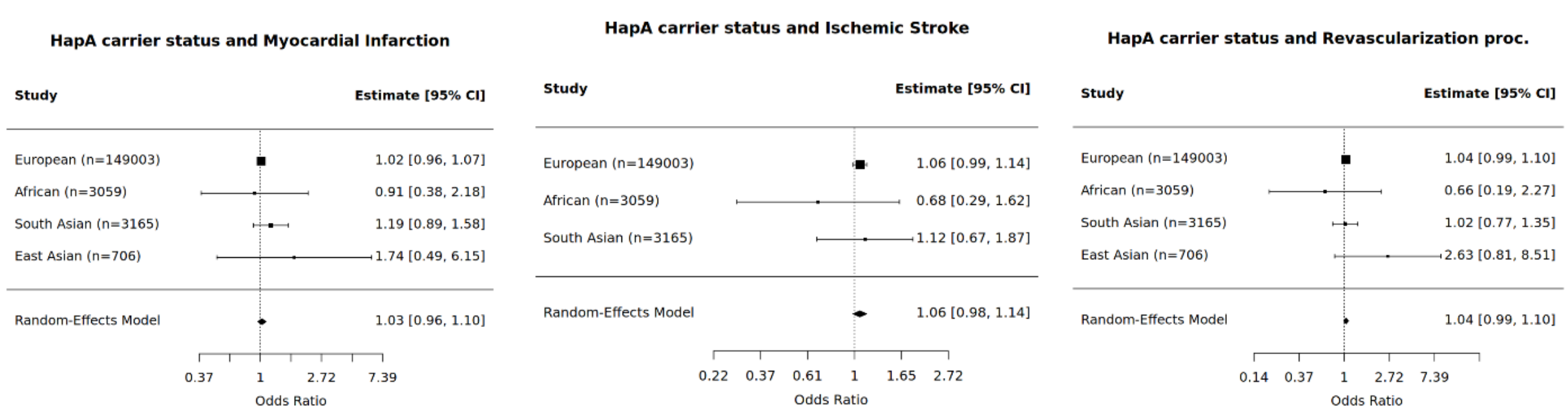


***Supplementary Figure 2: HapA dominant model and ASCVD outcomes***. *Random-effects trans-ancestry meta-analyses of odds ratios for ASCVD outcomes for carriers of haplotype HapA (rs17222814 (G), rs10507391 (T), rs4769874 (G), rs9551963 (A)). Models adjusted for age, age$^2$, sex, and 10 genetic principal components. East Asian ancestry was omitted due to insufficient number of cases for ischemic stroke.*

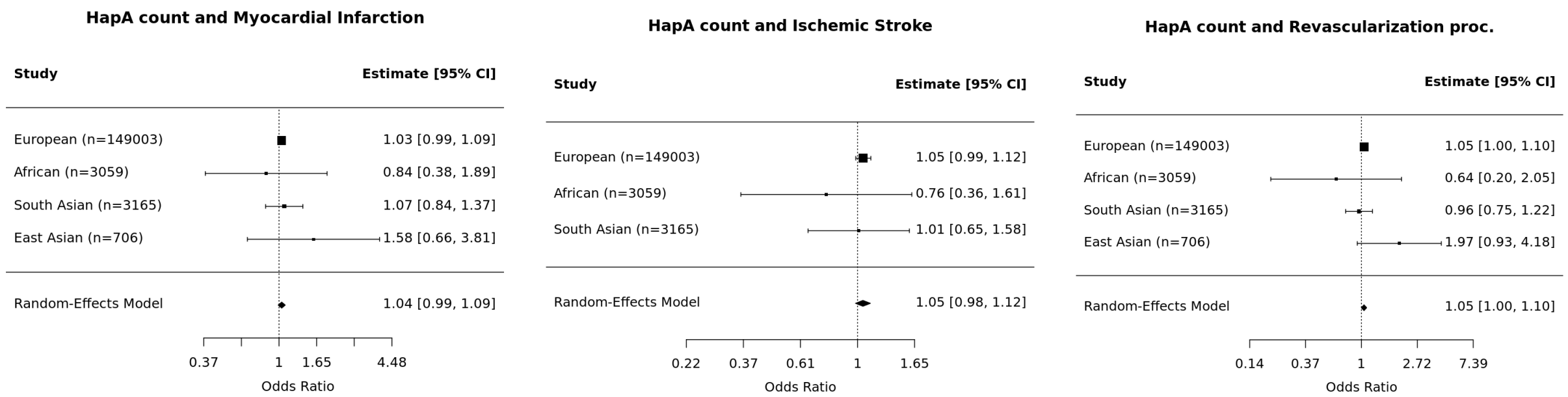


***Supplementary Figure 3: HapA additive model and ASCVD outcomes****. Random-effects trans-ancestry meta-analyses of odds ratios for ASCVD outcomes per copy of haplotype HapA (rs17222814 (G), rs10507391 (T), rs4769874 (G), rs9551963 (A)). Models adjusted for age, age*$^2$*, sex, and 10 genetic principal components. East Asian ancestry was omitted due to insufficient number of cases for ischemic stroke.*

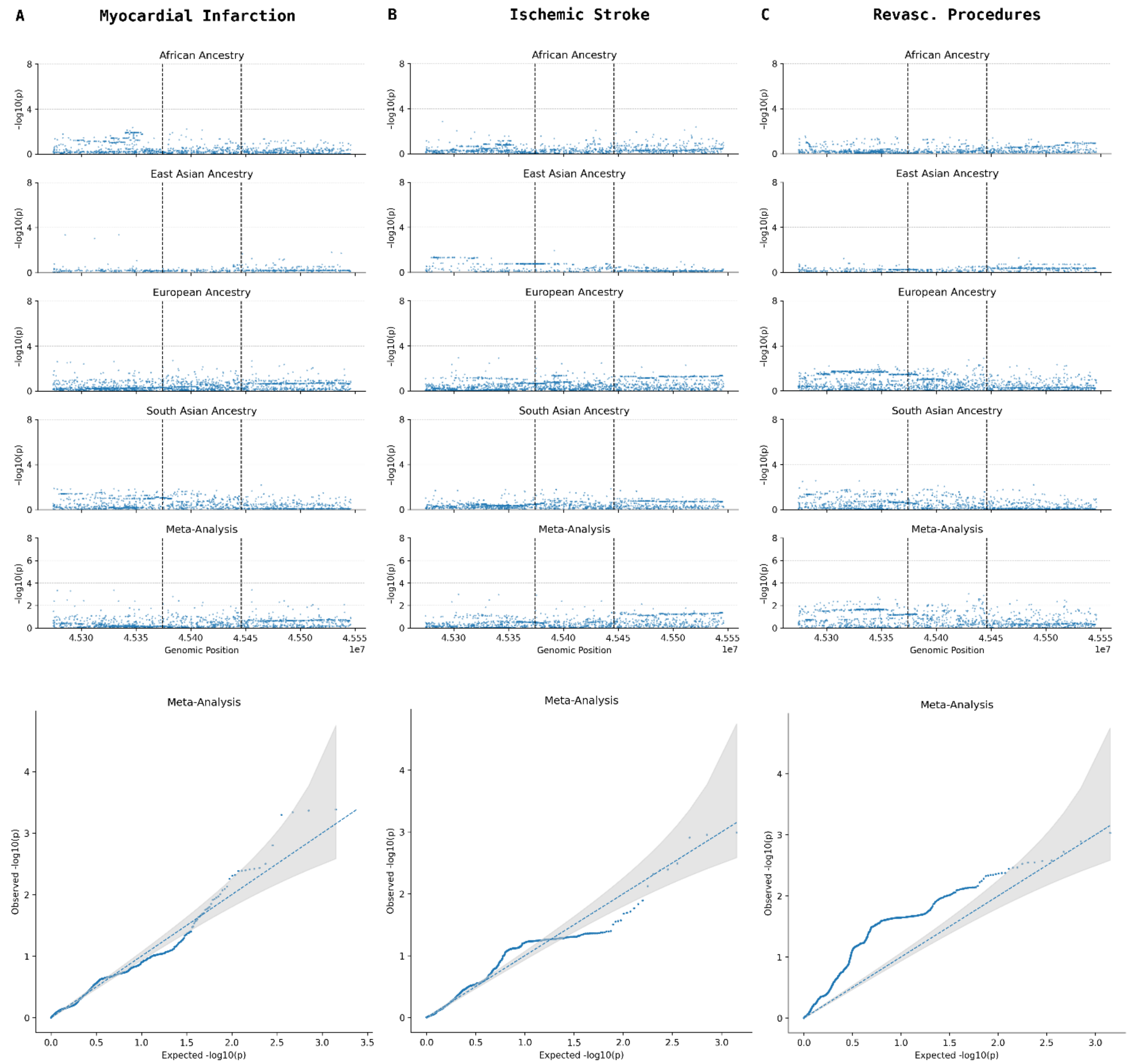


***Supplementary Figure 4: ALOX5 region single nucleotide polymorphism associations with coronary artery disease outcomes***. *Region plots of associations with binary traits generated with REGENIE for each ancestry and meta-analyzed using inverse-variance weighting. Under each set of region plots, there is a QQ plot of the meta-analysis for each outcome. Myocardial infarction (panel A), Ischemic stroke (panel B), revascularization procedures (panel C). Only variants present in at least two ancestries and with a global minor allele frequency of at least 0.001 were included in the meta-analyses.*

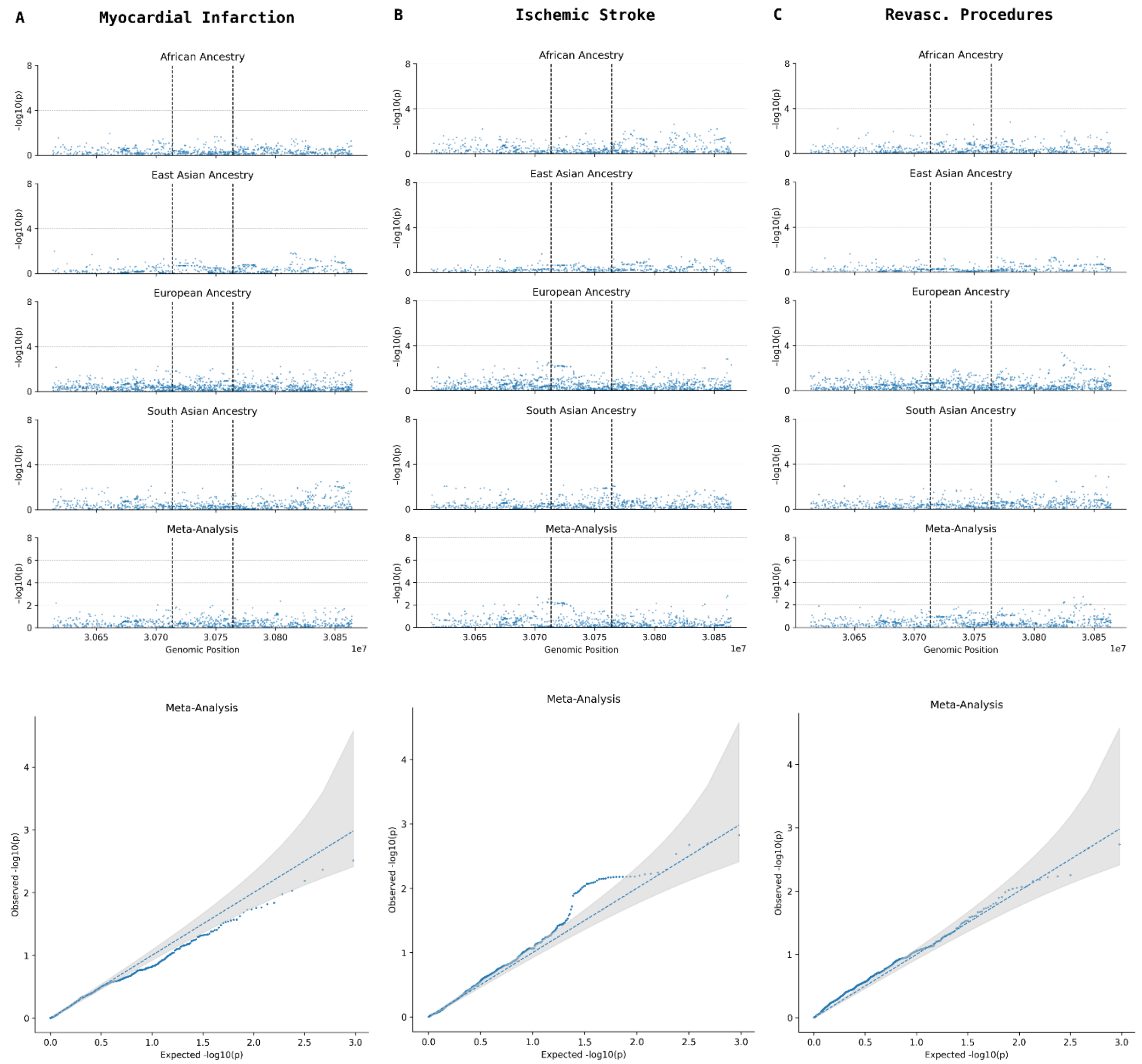


***Supplementary Figure 5: ALOX5AP region single nucleotide polymorphism associations with coronary artery disease outcomes****. Region plots of associations with binary traits generated with REGENIE for each ancestry and meta-analyzed using inverse-variance weighting. Under each set of region plots, there is a QQ plot of the meta-analysis for each outcome. Myocardial infarction (panel A), Ischemic stroke (panel B), Revascularization procedures (panel C). Only variants present in at least two ancestries and with a global minor allele frequency of at least 0.001 were included in the meta-analyses.*
