# Supplementary material for: Revisiting the genetic support of leukotriene pathway drug targets in atherosclerotic cardiovascular disease
Source: Front Genet. 2026 Oct 14;17:1812905. doi: 10.3389/fgene.2026.1812905 (PMC13713012; doi:10.3389/fgene.2026.1812905)
Supplement: Supplementary file 2 [file Table2.docx]

**Supplementary Table 2**: **UK Biobank data-fields**.

| **UK Biobank Data-field** | **Description** |
| --- | --- |
| [24310](https://biobank.ctsu.ox.ac.uk/crystal/field.cgi?id=24310) | DRAGEN whole genome sequencing .vcf files. Used to find Sp1 motif repeat number genotypes. |
| [20279](https://biobank.ctsu.ox.ac.uk/crystal/field.cgi?id=20279) | GATK and GraphTyper phased whole genome sequencing (200k subset). Used to find haplotype zygosity for analyses involving *ALOX5AP*. |
| [22671](https://biobank.ctsu.ox.ac.uk/crystal/field.cgi?id=22671), [22674](https://biobank.ctsu.ox.ac.uk/crystal/field.cgi?id=22674), [22677](https://biobank.ctsu.ox.ac.uk/crystal/field.cgi?id=22677), [22680](https://biobank.ctsu.ox.ac.uk/crystal/field.cgi?id=22680) | Baseline carotid intima medial thickness (cIMT) measurements that were averaged to obtain participant cIMT for use in analyses. |
| [31](https://biobank.ctsu.ox.ac.uk/crystal/field.cgi?id=31), [22001](https://biobank.ctsu.ox.ac.uk/crystal/field.cgi?id=22001) | Sex (reported) and sex (genetically determined), respectively. Samples were filtered to only retain those with matching reported sex and genetically determined sex as is standard to minimize participant-sample mismatch. Sex was also used as a covariate for analyses. |
| [21022](https://biobank.ctsu.ox.ac.uk/crystal/field.cgi?id=21022) | Age (instance 0). Used as a covariate for analyses. |
| [20002](https://biobank.ndph.ox.ac.uk/ukb/field.cgi?id=20002) | Non-cancer illness code, self-reported. |
| [22009](https://biobank.ctsu.ox.ac.uk/crystal/field.cgi?id=22009), [22020](https://biobank.ctsu.ox.ac.uk/crystal/field.cgi?id=22020) | 22009: 40 genetic principal components of individuals in UK Biobank. Used to obtain 10 first principal components to use as covariates. 22020: Analyses were restricted to those with “Used in genetic principal components” flag to exclude related individuals. |
| [50](https://biobank.ctsu.ox.ac.uk/crystal/field.cgi?id=50) | Standing height (instance 0). Used as a covariate for cIMT to replicate analyses previously done in the literature. |
| [21000](https://biobank.ctsu.ox.ac.uk/crystal/field.cgi?id=21000) | Self-reported ethnicity. Used to form clusters of genetically similar individuals (as determined by 10 first genetic principal components) to form ancestry groups. |
| [30710](https://biobank.ctsu.ox.ac.uk/crystal/field.cgi?id=30710), [30780](https://biobank.ctsu.ox.ac.uk/crystal/field.cgi?id=30780) | C-reactive protein and LDL-C levels (instance 0), respectively. Used for replication purposes and for higher sensitivity phenotypes to assess effects of *ALOX5* Sp1 motif variants. |
| [23445](https://biobank.ndph.ox.ac.uk/showcase/field.cgi?id=23445) | Plasma omega-6 measured by NMR metabolomics (generated by Nightingale Health). Used as higher-sensitivity molecular phenotype for *ALOX5* Sp1 motif variant effect. |
| [26227](https://biobank.ctsu.ox.ac.uk/crystal/field.cgi?id=26227) | Standard polygenic risk score for coronary artery disease. Used standard vs enhanced score to minimize sample loss (available n=485923 for standard vs available n=148835 for enhanced). The score was used as a sensitivity analysis for *ALOX5* and *ALOX5AP* to assess for a different effect of leukotriene pathway genetic variants in individuals genetically predisposed to coronary artery disease. |
