# Supplementary material for: Revisiting the genetic support of leukotriene pathway drug targets in atherosclerotic cardiovascular disease
Source: Front Genet. 2026 Oct 14;17:1812905. doi: 10.3389/fgene.2026.1812905 (PMC13713012; doi:10.3389/fgene.2026.1812905)
Supplement: Supplementary file 3 [file Table3.docx]

**Supplementary Table 3: Definitions of coronary artery disease-related clinical outcomes based on UK Biobank diagnostic and operation codes.**

| **Outcome** | **Code vocabulary** | **Code** | **Label** |
| --- | --- | --- | --- |
| **Myocardial infarction** | **ICD9** | **410** | **Acute myocardial infarction** |
|  |  | **412** | **Old myocardial infarction** |
|  |  | **411.0** | **Postmyocardial infarction syndrome** |
|  | **ICD10** | **I21** | **Acute myocardial infarction** |
|  |  | **I22** | **Subsequent myocardial infarction** |
|  |  | **I23** | **Certain current complications following acute myocardial infarction** |
|  |  | **I25.2** | **Old myocardial infarction** |
| **Revascularization (PCI/CABG)** | **OPCS** | **K40** | **Saphenous vein graft replacement of coronary artery** |
|  |  | **K41** | **Other autograft replacement of coronary artery** |
|  |  | **K42** | **Allograft replacement of coronary artery** |
|  |  | **K43** | **Prosthetic replacement of coronary artery** |
|  |  | **K44** | **Other replacement of coronary artery** |
|  |  | **K45** | **Connection of thoracic artery to coronary artery** |
|  |  | **K46** | **Other bypass of coronary artery** |
|  |  | **K49** | **Transluminal balloon angioplasty of coronary artery** |
|  |  | **K50** | **Other therapeutic transluminal operations on coronary artery** |
|  |  | **K75** | **Percutaneous transluminal balloon angioplasty and insertion of stent into coronary artery** |
| **Ischemic stroke (IS)** | **ICD9** | **434** | **Occlusion of cerebral arteries** |
|  |  | **434.0** | **Cerebral thrombosis** |
|  |  | **434.1** | **Cerebral embolism** |
|  |  | **434.9** | **Cerebral artery occlusion, unspecified** |
|  |  | **436** | **Acute, but ill-defined, cerebrovascular disease** |
|  | **ICD10** | **I63** | **Cerebral infarction** |
|  |  | **I64** | **Stroke, not specified as haemorrhage or infarction** |
