# Supplementary material for: Revisiting the genetic support of leukotriene pathway drug targets in atherosclerotic cardiovascular disease
Source: Front Genet. 2026 Oct 14;17:1812905. doi: 10.3389/fgene.2026.1812905 (PMC13713012; doi:10.3389/fgene.2026.1812905)
Supplement: Supplementary file 4 [file Table4.docx]

**Supplementary Table 4: ALOX5 promoter Sp1 motif Homozygous Non-5 Repeats Model**. Effect of carrying two European minor alleles at the ALOX5 promoter Sp1 motif variant (5 copies) vs having at least one European major allele on biomarkers and ASCVD outcomes in the UK Biobank. We report ancestry-specific effect estimates and a random-effects meta-analysis. Linear and logistic regressions were adjusted for age, sex, and first 10 genetic principal components. The linear model for carotid intima-media thickness was additionally adjusted for height. The association model seeks to replicate the findings from Dwyer et al.

|  | **Ancestry** | | | | |
| --- | --- | --- | --- | --- | --- |
|  | **European** | **African** | **South Asian** | **East Asian** | **Combined** |
| **Outcomes**  (OR [95% CI]) |  | | | | |
| **Myocardial Infarction** | 1.01 [0.93, 1.10], p=0.76 | 0.82 [0.60, 1.11], p=0.19 | 1.03 [0.75, 1.43], p=0.84 | 1.46 [0.60, 3.56], p=0.40 | 1.00 [0.93, 1.08], p=0.95 |
| **Revascularization Procedures** | 1.00 [0.92, 1.10], p=0.94 | 1.16 [0.78, 1.73], p=0.47 | 1.09 [0.80, 1.48], p=0.60 | 1.33 [0.52, 3.37], p=0.55 | 1.02 [0.94, 1.11], p=0.68 |
| **Ischemic Stroke** | 1.04 [0.92, 1.17], p=0.55 | 1.07 [0.78, 1.47], p=0.68 | 0.59 [0.27, 1.26], p=0.17 | 1.76 [0.62, 4.96], p=0.29 | 1.04 [0.93, 1.16], p=0.54 |
| **Biomarkers of ASCVD and inflammation**  (Mean difference [95% CI]) |  | | | | |
| **Carotid Intima-Media Thickness (μm)** | 3.09 [-3.70, 9.89], p=0.37 | -25.3 [-57.4, 6.71], p=0.12 | -45.8 [-98.0, 6.41], p=0.09 | 74.4 [19.3, 129], p=0.01 | -0.11 [-45.0, 44.8], p=1 |
| **LDL-C (mmol/L)** | 0.02 [-0.00, 0.03], p=0.08 | 0.02 [-0.02, 0.06], p=0.32 | 0.02 [-0.06, 0.10], p=0.64 | -0.04 [-0.15, 0.06], p=0.44 | 0.02 [-0.00, 0.03], p=0.05 |
| **C-reactive Protein (mg/L)** | -0.01 [-0.09, 0.08], p=0.88 | -0.35 [-0.58, -0.12], p=0.0030 | -0.12 [-0.48, 0.25], p=0.53 | 0.24 [-0.14, 0.62], p=0.22 | -0.08 [-0.30, 0.15], p=0.50 |
