# Supplementary material for: Revisiting the genetic support of leukotriene pathway drug targets in atherosclerotic cardiovascular disease
Source: Front Genet. 2026 Oct 14;17:1812905. doi: 10.3389/fgene.2026.1812905 (PMC13713012; doi:10.3389/fgene.2026.1812905)
Supplement: Supplementary file 5 [file Table5.docx]

**Supplementary Table 5: Association of HapA homozygosity in UK Biobank participants of European ancestry with clinical parameters.** Parameters include carotid intima-media thickness (cIMT; μm), LDL cholesterol (LDL-C; mmol/L), plasma omega-6 (mmol/L), and C-reactive protein levels (CRP; mg/L). Models were adjusted for age, age^2^, sex, and first 10 genetic principal components.

|  | **β** | **[95% CI]** | **P-Value** |
| --- | --- | --- | --- |
| **cIMT** | -4.49 | [-12.76, 3.78] | 0.29 |
| **LDL-C** | 0.0032 | [-0.017, 0.023] | 0.76 |
| **Omega-6** | 0.013 | [-0.0073, 0.034] | 0.21 |
| **CRP** | -0.031 | [-0.13, 0.072] | 0.56 |
