# Supplementary material for: Revisiting the genetic support of leukotriene pathway drug targets in atherosclerotic cardiovascular disease
Source: Front Genet. 2026 Oct 14;17:1812905. doi: 10.3389/fgene.2026.1812905 (PMC13713012; doi:10.3389/fgene.2026.1812905)
Supplement: Supplementary file 6 [file Table6.docx]

**Supplementary Table 6: ALOX5 promoter Sp1 variants, ALOX5AP’s HapA and Incident ACSVD.** Logistic regressions adjusted for age, age^2^, sex, and 10 first genetic principal components were fit using genetic data from individuals of European ancestry in the UK Biobank with self-reported or diagnosed ACSVD at date of consent (n=24188).

| **Exposure** | **β** | **95% CI** | **P-Value** |
| --- | --- | --- | --- |
| *ALOX5* | | | |
| Sp1 Motif Count | 0.01 | [-0.037, 0.057] | 0.68 |
| 2 Alternate Alleles for Sp1 Motif Count | -0.007 | [-0.16, 0.15] | 0.93 |
| *ALOX5AP* | | | |
| 2 Copies of HapA | 0.054 | [-0.14, 0.24] | 0.58 |
