# Supplementary material for: Revisiting the genetic support of leukotriene pathway drug targets in atherosclerotic cardiovascular disease
Source: Front Genet. 2026 Oct 14;17:1812905. doi: 10.3389/fgene.2026.1812905 (PMC13713012; doi:10.3389/fgene.2026.1812905)
Supplement: Supplementary file 7 [file Table7.docx]

***Supplementary Table 7: Significant variants in regions of ALOX5 and ALOX5AP for atherosclerotic clinical outcomes according to pre-established threshold***

| **Gene** | **Outcome** | **Variant ID** | **Meta Beta** | **Meta P-value** | **Ancestries** | **CARDIoGRAMplusC4D Beta** | **CARDIoGRAMplusC4D P-value** |
| --- | --- | --- | --- | --- | --- | --- | --- |
| ***ALOX5*** | MI | 10:45278142:A:C | 0.207 | 6.47x10^-4^ | AFR, EAS, EUR, SAS | 0.003 | 0.954 |
|  | MI | 10:45290209:G:A | 0.206 | 7.11 x10^-4^ | AFR, EAS, EUR, SAS | - | - |
|  | MI | 10:45302374:T:G | 0.327 | 1.07 x10^-3^ | EUR, SAS | - | - |
|  | MI | 10:45329936:T:G | 0.207 | 6.39 x10^-4^ | AFR, EAS, EUR, SAS | 0.022 | 0.741 |
|  | MI | 10:45455885:G:A | 0.181 | 9.93 x10^-4^ | AFR, EAS, EUR, SAS | -0.005 | 0.920 |

Variants from each ancestry were meta-analyzed using inverse-variance weighting. Only variants present in at least two ancestries, a global minor allele frequency of at least 0.001, and INFO score ≥ 0.80 were counted as significant hits. Only *ALOX5* had significant hits, and for MI only. External beta and p-values were taken from the independent cohort of CARDIoGRAMplusC4D 1000 Genomes-based GWAS evaluating MI with an additive model. Variant IDs are comprised of chromosome number, genomic position in GRCh38, reference allele, then effect allele.
