# Supplementary material for: Revisiting the genetic support of leukotriene pathway drug targets in atherosclerotic cardiovascular disease
Source: Front Genet. 2026 Oct 14;17:1812905. doi: 10.3389/fgene.2026.1812905 (PMC13713012; doi:10.3389/fgene.2026.1812905)
Supplement: Supplementary file 8 [file Table8.docx]

***Supplementary Table 8: Results of Transcriptome-Wide Association Study for Coronary Artery Disease for Available Genes in Leukotriene Pathway***

|  | **Ensembl Gene ID** | **Gene** | **Effect Size** | **P-value** | **Prediction R^2^** | **Prediction P-value** | **# SNPs in model** |
| --- | --- | --- | --- | --- | --- | --- | --- |
| **Whole Blood** | ENSG00000103222.18 | ABCC1 | -0.012 | 0.903 | 0.115 | 0.000 | 23 |
|  | ENSG00000124574.14 | ABCC10 | -0.113 | 0.518 | 0.010 | 0.018 | 30 |
|  | ENSG00000108839.11 | ALOX12 | 0.117 | 0.368 | 0.013 | 0.008 | 33 |
|  | ENSG00000012779.10 | ALOX5 | 0.203 | 0.615 | 0.025 | 0.000 | 20 |
|  | ENSG00000132965.9 | ALOX5AP | -0.105 | 0.231 | 0.198 | 0.000 | 16 |
|  | ENSG00000186204.14 | CYP4F12 | -0.011 | 0.769 | 0.212 | 0.000 | 57 |
|  | ENSG00000186529.15 | CYP4F3 | 0.107 | 0.335 | 0.051 | 0.000 | 20 |
|  | ENSG00000100031.18 | GGT1 | -0.052 | 0.551 | 0.085 | 0.000 | 24 |
|  | ENSG00000131067.16 | GGT7 | 0.247 | 0.179 | 0.074 | 0.000 | 32 |
|  | ENSG00000111144.9 | LTA4H | 0.035 | 0.488 | 0.124 | 0.000 | 27 |
|  | ENSG00000085871.8 | MGST2 | 0.032 | 0.600 | 0.184 | 0.000 | 49 |
|  | ENSG00000143198.12 | MGST3 | -0.069 | 0.098 | 0.284 | 0.000 | 8 |
|  | ENSG00000158517.13 | NCF1 | 0.026 | 0.818 | 0.058 | 0.000 | 16 |
|  | ENSG00000116711.9 | PLA2G4A | -0.072 | 0.802 | 0.011 | 0.015 | 8 |
|  | ENSG00000106853.18 | PTGR1 | 0.047 | 0.570 | 0.045 | 0.000 | 12 |
| **Artery (coronary)** | ENSG00000012779.10 | ALOX5 | 0.072 | 0.653 | 0.097 | 0.000 | 30 |
|  | ENSG00000186204.14 | CYP4F12 | -0.047 | 0.390 | 0.065 | 0.001 | 52 |
|  | ENSG00000106853.18 | PTGR1 | -0.006 | 0.901 | 0.090 | 0.000 | 106 |
| **Artery (aorta)** | ENSG00000108839.11 | ALOX12 | 0.048 | 0.510 | 0.054 | 0.000 | 13 |
|  | ENSG00000012779.10 | ALOX5 | 0.049 | 0.850 | 0.028 | 0.003 | 9 |
|  | ENSG00000132965.9 | ALOX5AP | -0.074 | 0.408 | 0.073 | 0.000 | 25 |
|  | ENSG00000187048.12 | CYP4A11 | -0.239 | 0.192 | 0.014 | 0.036 | 10 |
|  | ENSG00000186204.14 | CYP4F12 | -0.194 | 0.246 | 0.052 | 0.000 | 11 |
|  | ENSG00000100031.18 | GGT1 | -0.024 | 0.595 | 0.231 | 0.000 | 15 |
|  | ENSG00000099998.17 | GGT5 | 0.011 | 0.930 | 0.022 | 0.006 | 20 |
|  | ENSG00000131067.16 | GGT7 | 0.378 | 0.015 | 0.057 | 0.000 | 12 |
|  | ENSG00000085871.8 | MGST2 | 0.085 | 0.223 | 0.098 | 0.000 | 37 |
|  | ENSG00000143198.12 | MGST3 | -0.033 | 0.797 | 0.082 | 0.000 | 72 |
|  | ENSG00000127472.10 | PLA2G5 | 0.084 | 0.074 | 0.135 | 0.000 | 8 |
|  | ENSG00000106853.18 | PTGR1 | 0.012 | 0.632 | 0.313 | 0.000 | 78 |
