# Supplementary material for: Revisiting the genetic support of leukotriene pathway drug targets in atherosclerotic cardiovascular disease
Source: Front Genet. 2026 Oct 14;17:1812905. doi: 10.3389/fgene.2026.1812905 (PMC13713012; doi:10.3389/fgene.2026.1812905)
Supplement: Supplementary file 9 [file Table9.docx]

**Supplementary Table 9: Rare variant associations for ALOX5 and ALOX5AP gene regions found in Genebass and All by All data browsers.** Significance thresholds are from Bonferroni-corrected thresholds based on number of analzyes phenotypes. For All by All, the default maximum of 0.1% minor allele frequency was used.

| **Gene** | **Database** | **Statistical Test** | **Significant phenotypes** |
| --- | --- | --- | --- |
| **ALOX5** | Genebass | Burden (pLoF) | - |
|  |  | Burden (missense) | - |
|  |  | SKAT (pLoF) | - |
|  |  | SKAT (missense) | - |
|  |  | SKAT-O (pLoF) | - |
|  |  | SKAT-O (missense) | S66.2 Repair of nail bed, Oesophageal cancer, Y27.1 Autograft to organ NOC |
|  | All by All | Burden (pLoF) | Sprains and strains of hip and thigh, Levonorgestrel and Ethinylestradiol, Progestogens and Estrogens, Sequential Preparations, Fibrinogen, Opium Derivatives and Expectorants, Pancreatitis |
|  |  | Burden (missense) | - |
|  |  | SKAT (pLoF) | Sprains and strains of hip and thigh, Pancreatitis |
|  |  | SKAT (missense) | - |
|  |  | SKAT-O (pLoF) | Sprains and strains of hip and thigh, Pancreatitis |
|  |  | SKAT-O (missense) |  |
| **ALOX5AP** | genebass | Burden (pLoF) | - |
|  |  | Burden (missense) | - |
|  |  | SKAT (pLoF) | - |
|  |  | SKAT (missense) | E04 Other non-toxic goitre, Colposcopy +/- biopsy |
|  |  | SKAT-O (pLoF) | - |
|  |  | SKAT-O (missense) | E04 Other non-toxic goitre |
|  | All by All | Burden (pLoF) | - |
|  |  | Burden (missense) | - |
|  |  | SKAT (pLoF) | - |
|  |  | SKAT (missense) | - |
|  |  | SKAT-O (pLoF) | - |
|  |  | SKAT-O (missense) | - |
